# Breastfeeding, first-food systems and corporate power: a case study on the market and political practices of the transnational baby food industry and public health resistance in the Philippines

**DOI:** 10.1186/s12992-021-00774-5

**Published:** 2021-10-26

**Authors:** Phillip Baker, Paul Zambrano, Roger Mathisen, Maria Rosario Singh-Vergeire, Ana Epefania Escober, Melissa Mialon, Mark Lawrence, Katherine Sievert, Cherie Russell, David McCoy

**Affiliations:** 1 Institute for Physical Activity and Nutrition, School of Exercise and Nutrition Sciences, Deakin University, Geelong, Australia; 2 Alive & Thrive Southeast Asia, FHI 360, Manila, Philippines; 3 Alive & Thrive Southeast Asia, FHI 360, Hanoi, Vietnam; 4 Department of Health, Manila, Philippines; 5 School of Business, Trinity College Dublin, Dublin, Ireland; 6 School of Exercise and Nutrition Sciences, Deakin University, Geelong, Australia; 7 International Institute for Global Health, United Nations University, Kuala Lumpur, Malaysia

**Keywords:** Infant formula, Breast milk substitutes, Commercial determinants of health, Breastfeeding, Political economy

## Abstract

**Background:**

The aggressive marketing of breastmilk substitutes (BMS) reduces breastfeeding, and harms child and maternal health globally. Yet forty years after the World Health Assembly adopted the International Code of Marketing of Breast-milk Substitutes (The Code), many countries are still to fully implement its provisions into national law. Furthermore, despite The Code, commercial milk formula (CMF) markets have markedly expanded. In this paper, we adopt the Philippines as a case study to understand the battle for national Code implementation. In particular, we investigate the market and political strategies used by the baby food industry to shape the country’s ‘first-food system’, and in doing so, promote and sustain CMF consumption. We further investigate how breastfeeding coalitions and advocates have resisted these strategies, and generated political commitment for a world-leading breastfeeding policy framework and protection law (the ‘Milk Code’). We used a case study design and process tracing method, drawing from documentary and interview data.

**Results:**

The decline in breastfeeding in the Philippines in the mid-twentieth Century associated with intensive BMS marketing via health systems and consumer advertising. As regulations tightened, the industry more aggressively promoted CMFs for older infants and young children, thereby ‘marketing around’ the Milk Code. It established front groups to implement political strategies intended to weaken the country’s breastfeeding policy framework while also fostering a favourable image. This included lobbying government officials and international organizations, emphasising its economic importance and threats to foreign investment and trade, direct litigation against the government, messaging that framed marketing in terms of women’s choice and empowerment, and forging partnerships. A resurgence in breastfeeding from the mid-1980s onwards reflected strengthening political commitment for a national breastfeeding policy framework and Milk Code, resulting in-turn, from collective actions by breastfeeding coalitions, advocates and mothers.

**Conclusion:**

The Philippines illustrates the continuing battle for worldwide Code implementation, and in particular, how the baby food industry uses and adapts its market and political practices to promote and sustain CMF markets. Our results demonstrate that this industry’s political practices require much greater scrutiny. Furthermore, that mobilizing breastfeeding coalitions, advocacy groups and mothers is crucial to continually strengthen and protect national breastfeeding policy frameworks and Code implementation.

**Supplementary Information:**

The online version contains supplementary material available at 10.1186/s12992-021-00774-5.

## Introduction

The World Health Organization (WHO) recommends infants initiate breastfeeding in the first hour of life, exclusively breastfeed for 6 months, and thereafter receive nutritionally adequate and safe complementary foods, while breastfeeding continues for up to 2 years of age or beyond [1]. Yet, less than half of the world’s children meet these three recommendations [2, 3]. One key explanation for these low worldwide breastfeeding rates, is the aggressive marketing of breastmilk substitutes (BMS) [4]. Commercial milk formulas (CMF) are the main type of BMS marketed and consumed worldwide by infants (ages 0–12 months) and young children (ages 13–36 months). This includes standard infant formula (for ages 0–6 months), follow-up infant formula (7–12 months), toddler or growing-up milks (13–36 months) and specialised formula (for specific disorders, diseases or medical conditions) categories. Irrespective of country context, exposure to the marketing of these products increases bottle-feeding, and reduces breastfeeding initiation, exclusivity and duration [5, 6].

The WHO/UNICEF Global Strategy for Infant and Young Child Feeding calls on governments to protect, promote and support breastfeeding, including through the adoption of The International Code of Marketing of Breast-Milk Substitutes (The Code) into national law [7]. Adopted in 1981, The Code was the first of its kind adopted under the auspices of the United Nations (UN) system, intended to regulate the harmful practices of the baby food industry, at a time of accelerating globalization and growth in the size and economic power of transnational corporations [8]. The Code was also a response to the mobilization of a transnational advocacy network, the International Baby Food Action Network, and allies within governments, WHO, UNICEF and others in the UN system [9]. Importantly, implementation and monitoring of The Code is supported by the UN Convention on the Rights of the Child, and its monitoring body the Committee on the Rights of the Child.

Yet, as we mark the 40th anniversary of The Code, there is still a long way to go towards its full worldwide implementation. The latest monitoring report found that although 136 (70%) of 194 reporting countries had adopted at least some provisions of The Code into national law, just 25 (13%) were considered substantially aligned, and 58 (30%) had adopted no provisions whatsoever [10]. Furthermore, CMF markets have massively expanded since 1981 – between 2005 and 2019 alone, world sales more than doubled from 1 to 2.2 million tonnes per annum [11]. Elsewhere we describe this transition to higher formula diets as reflecting transformations in the systems that provision foods and structure infant and young child feeding practices at the population level – what we call *first-foods systems* [12].

Growth in CMF consumption is occurring mainly in industrialising middle-income countries, home to the world’s largest populations, indicating an unprecedented change in infant and young child diets [13]. It is also occurring in the context of continuing economic globalization, including rapid growth in the size, transnational reach and consolidation of the baby food industry, with the majority of sales accruing to just a small number of ‘Big Formula’ corporations [14]. Despite these developments, surprisingly few studies have investigated what market and political strategies the baby food industry has used to expand, sustain and protect its CMF markets worldwide, with some exceptions [15–18]. Nor have studies described how other first-food system actors, among them governments, international organizations, civil society groups and mothers, have successfully resisted corporate power to protect, promote and support breastfeeding.

In this paper, we adopt the Philippines as a case study to illustrate the continuing battle for worldwide Code implementation. We chose the Philippines because of its early and comprehensive implementation of The Code, consolidated and well-established baby food industry, its active civil society and ease of access to documentary data and key informants. Furthermore, The Philippines was also influential in generating international support for the adoption of The Code in the first place, evidenced by the speech given by Dr. Navidad Clavano in 1978, at the now famous Kennedy Hearings in the United States (US) Senate. Today, it is recognised as a ‘lighthouse’ country, being one of only 25 countries whose Milk Code law and supporting policies are ‘substantially aligned’ with The Code [10], and closely watched by others across the Southeast Asia region [19].

We also chose the Philippines because of the immense opportunity breastfeeding presents for sustainable development. The country’s exclusive breastfeeding rate (< 6 months) sits at just 34%. Scaling-up breastfeeding to near universal levels would prevent the deaths of an estimated 9000 Filipino children and 1900 mothers annually, and a further three million cases of child diarrhoea and pneumonia, and 16,800 cases of child obesity [20]. This would potentially save >US$16 million in health system treatment costs related to reduced mortality and morbidity, and generate US$3.8 billion for the economy (1.05% of gross national income), through increasing children’s cognitive capacity and preventing premature deaths. It would further reduce families’ out-of-pocket healthcare expenditures, and divert the US$839 million families’ spend on BMS every year to basic needs, including healthcare and food [20].

In this paper, our aim is to describe and understand the market and political practices used by the baby food industry to shape the Philippine first-food system in ways that drive and sustain CMF consumption, including its organised resistance to implementation of the country’s Milk Code. We also seek to describe how the power of this industry has been resisted, to protect and continually strengthen the country’s breastfeeding policy framework.

## Methods

Given the complex and multi-variable nature of the topic under study, we adopted a theoretically guided case study design [21], and process tracing method [22, 23]. This involved several steps. First, describing the scope and setting of the case study; second, collecting data from documentary sources and key informant interviews; and finally, synthesising results. To help develop initial concepts, guide our data collection and organize the results, we were guided by a theoretical framework (outlined in Text S1), which we developed in earlier studies on first-food systems and corporate power [12, 14]. We did not place constraints on the time-period under study, but allowed for an emerging understanding of events.

### Scope and setting of the case study

The Philippines is an archipelago nation in Southeast Asia, spanning 7600 islands across three main geographical areas [24]. The population of 111 million includes diverse ethnic and cultural groupings [25]. It is a lower-middle income country, with a gross national income per capita of US$3850 in 2019 [26], with 16.6% of Filipinos living below the national poverty line in 2018 [27].

Since 1986, a presidential democratic constitutional republic system of government has been in place, comprising a bicameral Legislative Congress of the Senate and House of Representatives; a Judiciary, with the Supreme Court as the highest body; and an Executive, including the President and Cabinet [28]. Lawmaking requires a draft bill to be filed in and approved by both the Senate and House of Representatives respectively, before transmission to the President who may either sign the bill into law as a Republic Act, veto, or take no action – in which case the bill lapses into law after 30 days. The President may also issue orders, proclamations, or circulars under the ordinance powers granted by the Constitution, including Executive Orders that have the force and effect of law [29]. Government is strongly decentralized, with semi-autonomous Local Government Units (LGUs) at province, city, municipality and Barangay levels [30].

Links with the international system include membership in the United Nations (1945), World Trade Organization (1995) and Association of Southeast Asian Nations (1967). The Philippines has ratified numerous human rights treatises, including the Convention on the Rights of the Child (1990) and the Convention on the Elimination of All Forms of Discrimination Against Women (1981), [31]. The Western Pacific Regional Office of WHO is located in Manila, as are country offices of various international organizations. The Philippines was under Spanish colonial rule from the sixteenth Century, followed by the United States (US) in 1901. Close relations with the US persisted until the current administration, under President Rodrigo Duterte, ushered a recent foreign policy pivot towards China [32].

### Data collection

We applied standard case study data collection techniques, allowing us to triangulate across multiple data sources [22, 23]. First, we conducted focused searches of academic (Scopus, PubMed, Google Scholar) and internet (Google) databases to find journal articles, using infant and young child feeding (IYCF) related key words and search strings. As our understanding of the case study evolved, we conducted further branching searches, including focused searches on the two market leaders Nestlé and Mead Johnson (Reckitt Benckiser), and their corporate websites. From these initial documents, and from consultations with local experts, we identified other key actors. We then collected publicly available documents from the websites of government agencies, industry groups, professional associations, civil society organizations, and the media. To source archival website data, we used the Way Back Machine (Internet Archive; https://archive.org/web/).

To complement the documentary data, the lead author conducted interviews with 17 participants between May and November 2020 [33]. Participants were recruited using a purposive snowball sampling method [34], identified by their sector or study number only. Initial email invitations were sent to 32 individuals. Three declined to participate, all from industry, and 12 did not respond. Participants were from government (*n* = 3), civil society (*n* = 4), international organizations (*n* = 8), and academia (*n* = 2). All interviews were online, ranging from 30 to 90 min in duration. The interviews were semi-structured and followed an interview guide developed from the framework. With consent, interviews were recorded and transcribed. Several informants provided additional documentary evidence.

### Data analysis and reporting

Interview transcripts and documents were coded using NVivo by the lead author. A coding schema was developed from the theoretical framework (Text S1) and additional emergent themes were captured using open coding. The coding schema was refined using constant comparative thematic analysis. The final interpretation of events was clarified through ‘member-checking’ with three informants [33].

### Ethics, funding and reflexivity

This study was funded by the WHO’s Department of Maternal, Newborn, Child and Adolescent Health. The funder played no role in the conduct of the research, and all interpretation of the data and findings are the authors' alone. The research team drew from both academic and practitioner experience, including experts in infant and young child feeding, public health nutrition and food policy. This study was approved by Deakin University’s Human Research Ethics Committee (2019–398).

## Results

We structure the results as follows. First, we describe historical changes in the Philippine first-food system, including the role of BMS marketing, and associated changes in IYCF indicators. Second, we describe the political strategies used by the baby food industry to foster a policy, regulatory and knowledge environment that enables its marketing. Third, we describe how, despite this corporate political activity, political commitment for a comprehensive policy framework to protect, promote and support breastfeeding emerged in the country. Finally, we consider the power of breastfeeding coalitions, advocates and mothers.

### The Philippine first-food system, BMS marketing and the decline and resurgence of breastfeeding

The Philippines experienced a dramatic decline in breastfeeding in the mid to late twentieth century, followed by a resurgence. Table 1 presents data on relevant indicators for the period 1963–2017. Breastfeeding rates reached a historic low-point in the early-1980s. Between 1967 and 1983 the median duration of breastfeeding declined by 16% from 14.5 to 12.1 months [35], before steadily rising to 19.8 months in 2017. Other breastfeeding indicators reported from 1993 onwards, show a steady improvement in breastfeeding initiation, those ever-breastfed, and continued breastfeeding at 20–23 months. However, since 1993, the percentage of infants exclusively breastfed (< 6 months) fluctuated between 24.7 and 37.5%, and today remains well below the World Health Assembly's Global Target of 50% by 2025.

**Table 1:** Trends in demographic, infant and young child feeding, and maternal, neonatal and child health indicators in the Philippines, 1967–2017

Category	Indicators	1967	1972	1977	1982	1987	1993	1998	2003	2008	2013	2017
Demographic	Population (millions)^a^	32.8	37.9	43.6	50.0	57.3	66.6	74.7	83.1	90.9	98.9	105.2
	GNI per capita (constant 2010 US$)^a^	1240	1343	1612	1724	1407	1521	1845	1926	2298	2787	3369
	Urban population (%)^a^	32.1	34.0	36.3	39.3	44.2	46.7	46.3	45.9	45.5	45.9	46.7
	Female labor force participation (% aged 15+)^a^	–	–	–	–	48.3	47.8	49.3	46.9	46.9	48.3	44.9
	Birth rate (per 1000 people)^a^	40.6	38.5	37.4	35.9	34.2	31.8	30.1	28.4	25.8	23.6	21.0
Infant and young child feeding	Median breastfeeding duration (months)	14.5^c^	13.7^c^	12.9^c^	12.1^c^	12.5^d^	14.1^e^	12.8^e^	14.1^e^	14.3^e^	16.7^e^	19.8^e^
	Breastfeeding initiation (%)^b^	–	–	–	–	–	35.6	34.5	46.1	48.3	49.7	56.9
	Ever breastfed (%)^b^	–	–	–	–	–	88.1	89.1	86.8	89.6	93.7	93.2
	Exclusive breastfeeding 0–5 months (%)^b^	–	–	–	–	–	28.9	37.5	34	33	24.7^f^	29.0^f^
	Continued breastfeeding 20–23 months (%)^b^	–	–	–	–	–	24.3	29.5	32.3	34.4	40.9	52.3
	Percentage of baby friendly health facilities^g^	–	–	–	–	–	–	83	–	–	–	5
Maternal, neonatal and child health	Neonatal mortality rate (per 1000 births)^b^	–	25.5	25.2	24.0	21.4	17.8	16.5	16.0	15.2	14.6	13.9
	Infant mortality rate (per 1000 births)^b^	57.5	54.7	54.1	51.7	45.9	34.9	29.8	27.3	25.4	24	22.7
	Under-5 mortality rate (per 1000 births)^b^	87.6	82.7	81.7	77.4	67.1	47.8	39.4	35.6	32.6	30.6	28.7
	Number of under-5 child deaths (thousands)^b^	–	117.1	128.9	135.0	128.1	99.3	87.3	83.1	76.4	71.3	64.1
	Birth deliveries in a health facility (%)^b^	–	–	–	–	–	28	34	38	44	61	78
	Pregnancy attended at least once by SHP (%)^b^	–	–	–	–	–	83	86	86	88	91	95

*Notes*: ^a^ data from [26], ^b^ from [2], ^c^ from [35, 36], ^d^ from [37], ^e^ from [38], ^f^ from [39], data in the 2013 column is for 2015, and in the 2017 column for 2018, ^g^ from [40], data in the 2008 column is for 2009, and in the 2017 column for 2016, *SHP* Skilled health professional

These historical trends in breastfeeding rates are explained by changes in the Philippine first-food system. Several studies reported the decline in breastfeeding as occurring mainly among wealthier, urban and more educated mothers. This was attributed to income growth, social changes associated with urbanization and modernity, rising women’s workforce participation, the medicalization of birthing and newborn care (especially in urban private hospitals), and the widespread availability of BMS in the country, together with intensified commercial marketing through health systems and directly to consumers via retail channels [35, 37, 41, 42].

In 1981, a survey of 100 barangays (villages) in Bicol, the country’s third poorest region, found BMS widely distributed through birthing facilities and retail stores. Of the private and public hospitals surveyed, 95 and 67% had received free infant formula samples respectively, of which ~ 80% distributed these samples ‘all of the time’. Donations of equipment and supplies to health clinics was common with, for example, 95% of infant identification bracelets carrying a BMS manufacturer’s brand. In private and public hospitals, 28 and 20% respectively provided the names of mothers to sales representatives, or allowed direct contact in the facility itself [43].

A 1982 survey of staff in clinics across Metropolitan Manila, found 70% were visited by sales representatives (so-called ‘med-reps’) at least once per month, with a range of one to 20 visits [44]. Between 1984 and 1986 in Metropolitan Cebu, another study found over half of clinics were distributing BMS samples, with 40% allowing direct contact between sales representatives and mothers. In the 2 years following the passage of the Milk Code in 1986, these practices declined significantly, and in many clinics ceased altogether. However, the availability of formula for older infants in health facilities, a product category not covered by the Milk Code at the time, surged by 80% [45].

Between 2003 and 2005, total milk formula sales boomed from US$260 million to US$420 million [46], reached US$699 million by 2006, and then increased by a further 19% to US$839 million in 2020 [11]. Between 2006 and 2020, there were modest declines of − 6.1 and − 6.2% in infant and follow-up formula sales respectively, but an increase of 49 and 62% in toddler milk and specialised formula sales [11]. In 2003, a national survey found almost half of families with infants and young children purchased BMS, including one-third of families living on less than US$2 per day. This indicates BMS use was no longer limited to wealthier groups, but also common among the country’s poorest consumers [11].

Marketing expenditure data is sparse for the Philippines. However, between 2006 and 2007, an estimated ~US$85–100 million was spent on BMS advertising, about half the annual budget of the Department of Health [46]. The industry ranked sixth among the top ten advertising ‘big spenders’ in the country [47]. Such marketing is influential. In 2011, a survey study reported 59% of mothers with young children recalled having seen a CMF advertisement. The use of BMS was twice as likely among those with recall of an advertisement versus those without, and associated with a six-fold greater likelihood of breastfeeding cessation before 12 months of age [48].

As reported in many other countries, we found evidence of ‘medical marketing’ through health professionals. For example, in 2020, Nestlé, Abbott and Mead Johnson (Reckitt Benckiser) sponsored the Annual Convention of the Philippine Pediatric Society (PPS). The PPS wrote to the Department of Health stating that due to the Covid-19-related economic crisis ‘partnering with pharmaceutical and nutritional companies can help bring this medical forum to succeed’, and requested a ‘more understanding and supportive EO51 and Milk Code be implemented at this time’ [49]. Five of the 15 members of the PPS Board of Trustees, including the President and Secretary, declared a conflict of interest in their curriculum vitae, as a ‘Member Speakers Bureau’ or ‘Key Opinion Leader’ for a baby food / pharmaceutical company.

From 2017 and 2021, the industry also engaged other professionals on the use of infant formulas, follow-up formulas and human milk fortifiers. Among them were the Integrated Midwives Association of the Philippines, Philippine League of Government and Private Midwives, the Philippine Association of Nutrition and the Nutritionist Dietitians Association of the Philippines, as well as individual paediatricians in the conduct of health worker seminars and research dissemination. This included sponsorship for conference attendance, accommodation and travel, and sponsored scholarships and grants for graduate and post-graduate students [50].

### The structure and corporate political activity of the baby food industry in the Philippines

To grow and sustain its market in the Philippines, the baby food industry has not only made large investments in marketing, including inappropriate promotion. It has also implemented political strategies to prevent, delay or weaken  policy frameworks and regulations that constrain its marketing activities.

#### The structure of the Philippine baby food industry

The baby food industry has long been present in the Philippines. Milk formula use was reported in the early-twentieth century, when Filipino’s traveling to Europe were introduced to artificial feeding, and by 1907 had established the La Gota de Leche in Manila, as a philanthropic organization to distribute bottled milk to infants unable to breastfeed. By 1910, reports of diarrheal disease among bottle-fed infants appeared in the medical literature [41, 51].

Given the country’s high birth rate and large population, the Philippines represents an important market for the baby industry. For example, 2.5 million babies are born every year in the country, compared with just 300,000 in Cambodia [47]. Nestlé, the world’s largest food manufacturer, was at the vanguard of the industry’s first-wave of globalization in the late-1800s, hence benefiting from its ‘first-mover advantage’ in many markets. This included the Philippines, where it was marketing its products as early as 1895, and established its first permanent sales office in Calle Renta, Binondo in 1911 [52]. As of 2018, Nestlé reported employing 3700 people across the country [52].

**Fig. 1:**
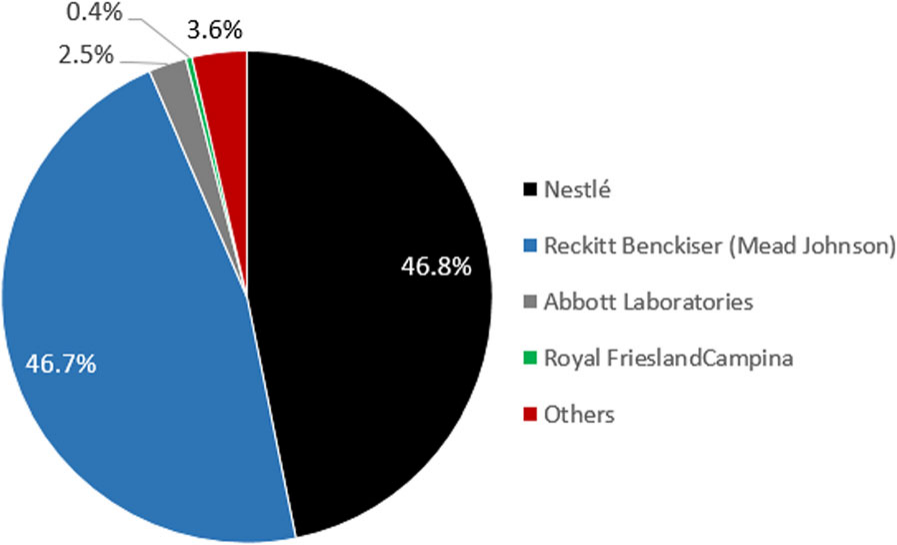
Philippines’s BMS market structure in 2020, showing % share of market leaders. Notes: Data from [11]

As Fig. 1 shows, the Philippine CMF market was strongly oligopolistic in 2020, dominated by a small number of transnational corporations. Nestlé and Reckitt Benckiser (UK; Mead Johnson) controlled 94% of sales between them, each with a 47% market share [11]. Abbott Laboratories (US) and Royal FrieslandCampina (RFC; Netherlands) are minor players with 2.5 and 0.4% shares respectively. Further indicating the importance of the market, Danone (France) recently entered the Philippines [53]. Other companies were key players until 2012, when RFC acquired the Filipino milk formula manufacturer Alaska Milk Corporation, and Nestlé acquired the long-standing market leader Wyeth (US).

Informants referred to three major recent periods of corporate political activity (CPA), which aligned closely with our documentary evidence.

#### First major period of corporate political activity (CPA), 2004–07 – attempts to weaken the Milk code

On September 27, 2004 the DOH initiated the drafting of Revised Implementing Rules and Regulations (RIRRs) to strengthen the country’s Milk Code. The stated purpose was “… to achieve the relevant constitutional mandates, implement international commitments and provide solutions to the problems identified in scientific and medical studies” and to respond to the “… undue advantage of the loopholes and gaps in the Milk Code and its previous implementing rules [taken by BMS manufacturers and distributors] to massively undermine breastfeeding in the Philippines” [54].

A technical working group was established to guide the process, comprising government, civil society and academic representatives, in consultation with UNICEF and WHO [54]. The planned RIRRs included inter alia adopting stronger administrative and criminal penalties for violators; restrictions on industry participation in breastfeeding policy development; and new prohibitions on health and nutrition claims on labels, pictures idealizing infants and children on products, gifts including free samples to health professionals, the provision of health professional training, and BMS donations during emergencies [55].

Public consultations were convened with interested parties on February 28 and July 8, 2005, both attended by milk formula manufacturers, and the latter by the Advertising Board of the Philippines and other industry groups, with various position papers submitted beforehand [56]. Despite this consultative process, the Pharmaceutical and Healthcare Association of the Philippines (PHAP), a lobby group representing the US pharmaceutical companies Abbott Ross, Mead Johnson, Wyeth and others [57], initiated a wide-reaching interference campaign, intended to block passage of the RIRRs.

This was first apparent when industry lobbyists, and then officials from the US Embassy and State Department, applied political pressure during meetings with the Secretary of Health and his undersecretaries [47]. The Secretary later stated;

They wanted the old provisions of the law [reinstated] … We could not agree. Those were the very loopholes that existed and allowed them to go around the [rules] … some very influential people [were] telling us [about] the economic implications, if these guys … close their operations and move out of the country. That might lead to unemployment [47]

Lobbying at the highest level soon followed. On January 12, 2006, the CEO of PHAP addressed a letter to the President of the Philippines Gloria Macapagal Arroyo, raising;

...questions and protests regarding the constitutionality, legality and validity of certain provisions … which make the [RIRRs] susceptible to a temporary restraining order and … the government to possible sanctions imposed by the World Trade Organization [58].

The letter claimed the RIRRs constituted an improper use of administrative power (that as subordinate legislation it goes beyond EO51); further, that the DOH lacked the legal authority to impose administrative sanctions; and that it violated trademark and other intellectual property protections of the WTO Agreement on Trade-Related Aspects of Intellectual Property Rights (TRIPS). The CEO urged the President to consider other causes of infant mortality, and to postpone implementation of the RIRR “until a committee is able to make extensive studies and refinements in the provisions” [58].

On January 20, 2006, PHAP representatives met the Secretary of Health, to further voice their concerns [56]. Ten days later, on January 30, the President of PHAP wrote a letter to the Chairman of the Committee on Trade and Industry, echoing many of the arguments made earlier to the President, stating “… the IRR will discourage investment consideration in the Philippines. Two of our members are poised for expansion of their facilities and have investment approval to proceed. However, some reservations are now being raised about the merit of proceeding” [59]. Furthermore, that PHAP;

… seek [s] your committee’s help in asking the Secretary of Health to defer signing and implementation [of the RIRRs] … until further evaluation can be made to assess its legality, constitutionality and impact to trade, employment and investment. We also believe that the interest of Filipino infants can best be served through an effective education campaign on proper nutrition directed to mothers [59]

In a letter dated the same day, the Department of Trade and Industry wrote to the Chairman of the Committee on Trade and Industry, stating that the RIRRs;

… may result in an infringement of the fundamental right of consumers to information and choice … Moreover, we believe that the policies of the State must be liberalized to give industry players, local or foreign, the right to promote their products within the scope of the Code. Otherwise, said restrictive provisions as cited herein might result to the damage of the infant formula sub-sector, which employs a substantial number of Filipinos [60]

Industry positions differed. The CEO and Chairman of Nestlé Philippines wrote to the Chairman’s of the Committee on Health and Committee on Trade and Industry, seeking to distance itself from PHAP;

… [the] position of Nestlé Philippines on the two bills is quite different from the position taken by members of [PHAP] … our company is not a member … [nor] involved in the positions taken and representations made [61]

Instead, Nestlé argued that the RIRRs should cover products for ages 0–12 months (consistent with its own corporate policy), but not complementary foods for infants aged over 6 months, nor growing up milks and other milk products for beyond 1 year of age. It opposed the prohibition of continuing medical education, scholarships and research grants, and the dissemination of scientific and educational materials to health professionals [61].

On February 16, a Congressional hearing was held where public health and industry representatives presented their respective positions on the RIRRs [56]. This was followed on March 27, by a letter sent from the milk manufacturers to the Secretary of Health [56]. In the spirit of compromise, the draft RIRRs were revised 19 times by the DOH over the 2 years under development, accommodating several industry requests, eventually signed for approval by the Secretary of Health on May 15, 2006.

Lobbying was against the RIRRs was then coordinated internationally. On June 30, 2006, the International Formula Council (IFC; later rebranded the Infant Nutrition Council of America), a US-based lobby group for the baby food industry, wrote a letter to UNICEF’s Director General Ann Veneman. This requested a meeting and claimed the breastfeeding promotion activities of UNICEF’s Philippines country office ‘misrepresents the available scientific evidence regarding the alleged risks of not breastfeeding’ [46, 62]. A letter was also sent to the UNICEF regional office in Bangkok, complaining about the ‘unscientific’ remarks and questioning the competency of the UNICEF-Philippines country representative. A similar complaint was made against the WHO country representative [63].

On July 11, 2006, PHAP followed through on the earlier threat made to the President, by filing a petition to the Supreme Court. This disputed the authority of the DOH and the constitutionality of certain provisions of the RIRRs, and requested the issuance of a Temporary Restraining Order (TRO) to block its implementation [46, 56]. In its submission, PHAP outlined the cost of compliance with the RIRRs at US$192 million, a rare example of the industry’s own estimates of the cost of regulatory compliance [64]. On July 11, the Supreme Court issued a denial of the petition, and on July 24th PHAP submitted a request for a reconsideration of the decision [56].

At this stage, the baby food industry leveraged its wider influence network of allied business interest groups. On August 1, the President of the US Chamber of Commerce (USCC), a trade association representing millions of US businesses, wrote to President Arroyo saying the RIRRs put US-Philippine trade relations into jeopardy. The letter stated the RIRRs;

… would have unintended negative consequences for investors’ confidence in the predictability of business law in the Philippines...If regulations are susceptible to amendment without due process, a country’s reputation as a stable and viable destination for investment is at risk [65, 66]

Furthermore, that;

… the IRR treats infant formula as a potential health hazard by requiring warning labels without any scientific justification, a step which would needlessly alarm potential consumers [65, 66]

The letter proposed USCC member companies were “… most willing to work with your Government to fashion a robust regulation to support consumers’ educational and health needs” [65, 66]. This was soon followed by a counter letter from the Secretary of Health, correcting several erroneous statements made by the USCC. On August 2, 2006, IFC lobbyists met with officers of the Philippine Embassy in Washington D.C., stating it supported the position of the PHAP, as outlined earlier in the position paper sent to President Arroyo [67]. Earlier that month, the President did not deliver her annual speech during World Breastfeeding Week celebrations [63]. Although we could not ascertain the exact date, photo evidence shows senior executives from Wyeth Philippines making a ‘courtesy call’ on President Arroyo at the Malacanang Palace during this period [68].

On August 15, 2006, the Supreme Court reversed its initial decision, and issued the requested TRO, effectively preventing the DOH from implementing the RIRRs. Although the DOH had filed an urgent motion to lift the TRO on September 6, 2006 [64], it was not until June 19, 2007, when the Supreme Court considered oral arguments on the case, and it only delivered its decision on October 9, 2007. It ruled partially in favor of PHAP, agreeing that a total prohibition on advertising of all products and the proposed administrative sanctions set out in the RIRRs, could be implemented only if a law was passed to amend the Milk Code. However, it ruled overwhelmingly in favor of the DOH and lifted the TRO, stating that all other provisions of the RIRRs were consistent with the objectives and purpose of the Milk Code, and justified to protect public health [54].

During this period, PHAP also initiated public influence campaigns. In November, 2006, it advertised in leading newspapers stating its support for breastfeeding as best for infants, but implied that breastfeeding advocates were limiting mother’s freedom of choice, and cited statistics that breastfeeding rates were also high in the Philippines, which UNICEF later reported as erroneous [69]. In February, and then during World Breastfeeding Week in August, 2007, it ran advertisements expressing concern for women unable to breastfeed, and again reported official breastfeeding statistics framed as ‘encouraging positive trends’ [70]. A New York based global strategic research firm also conducted an opinion poll that year, with the results released through a ‘Mother Knows Best’ group, emphasizing ‘the right to choose’ [71].

#### Second major period of CPA, 2007–13 – stealth legislation

This period of CPA focuses on the attempted passage of new legislation supported by the baby food industry. If passed, this would have significantly weakened the country’s breastfeeding policy framework, and especially the Milk Code.

On December 7 and 10, 2007, the DOH issued guidelines establishing mandatory labeling standards for BMS and related products within scope of the Milk Code [72, 73]. That same year the companies Abbott, Nestlé, Wyeth, Mead Johnson and Fonterra, established a new front group – the Infant and Pediatric Nutrition Association of the Philippines (IPNAP). In May 2009, they convened a Technical Work Group, including representatives of its member companies [74]. At the time of writing, IPNAP was governed by representatives from Abbott and Nestlé, and its Executive Director was a former Congressman and previous employee of the Office of the Secretary of Health [75]. The IPNAP website states it was formed;

… to establish the industry’s collective approach to improving nutrition [and serve] as the industry’s platform in promoting nutrition and development … and in advocating for government policies that enable the business community to demonstrate genuine corporate citizenship [75]

IPNAP is an active member of the Asia Pacific Infant and Young Child Nutrition Association [76], a regional industry lobby group, and the International Special Dietary Foods Industry (ISDI), the industry’s peak international trade association [75]. IPNAP participates in the Philippine delegation, and APIYCNA participates in various other member state delegations, to the Codex Alimentarius Commission, the UN food regulatory body, alongside ISDI, which represents the industry in Codex as an observer. Through ISDI, IPNAP connects with a global network of at least 20 ‘infant nutrition’ trade associations, representing milk formula manufacturers at international regional and country levels [77].

On September 5, 2011, on behalf of the IAC, the DOH issued a Memorandum directing its Bureau of Food and Drugs (FDA) to disallow “… any kind of trademarks” on all products covered by the Milk Code “… that contain health and nutrition claims or that may undermine breastfeeding and breastmilk to be placed on the labels” with the justification “that labels are marketing materials”. According to the IAC, companies were attempting to legitimise various marks and claims for use on their products, by registering them with the Intellectual Property Office of the Philippines, and the “total effect” undermined breastfeeding, by portraying products as equivalent with, or as superior to, breastmilk [78].

In response, IPNAP wrote a 13-page letter protesting the Memorandum. It called on the IAC to consider the “far reaching effects” on the intellectual property rights of its members. The letter questioned the legality of the Memorandum, referring to the Intellectual Property Code of the Philippines, and the WTO’s Technical Barriers to Trade (TBT) and TRIPS Agreements. Faced with this challenge, the DOH sought legal advice from the Department of Justice. In May and September 2012, Justice Secretary Leila de Lima issued two respective legal opinions reaffirming the legality of the Memorandum, and the FDA’s legal authority to prohibit the use of registered trademarks [79, 80]. Secretary de Lima stated;

IPNAP member companies, in the exercise of their property rights, also have a responsibility to the public. Just because they have the marks containing health and nutrition claims trademarked does not mean that their use cannot be regulated for the greater good [79]

On September 10, 2012, the DOH issued a second Memorandum, directing the FDA to strictly enforce the established trademark restrictions, inclusive of a list of restricted brand names [81].

Throughout this period there is evidence of IPNAP lobbying. In July 2010, for example, at the start of the 15th Congress, IPNAP met with the Vice-Chair of the Committee on Health, the Speaker of the House, various legislators and members of the Senate [82]. In 2012, four new draft House Bills (HB) were filed in the House of Commons, and in May that year, consolidated into a single Bill entitled “An Act Promoting a Comprehensive Program on Breastfeeding Practices and regulating the Trade, Marketing and promotions of Certain Foods for Infants and Children” [83]. Three of the draft Bills – HB3525 (Rep. Gunigundo), HB3527 (Reps. Lacson-Noel and Rodriguez), and HB3537 (Reps. Mercado-Revilla and Torres-Gomez) – contained provisions favourable to industry [84].

In a position paper, IPNAP framed the Bill as a ‘progressive piece of legislation’ [85]. Breastfeeding advocates, on the other hand, labelled it the ‘Milk Monster Bill’. If passed, it would have substantially weakened the Milk Code, the Expanded Breastfeeding Act (2009), and their respective IRRs, including among other things narrowing the product scope to 0–6 months; allowing donations during emergencies with the approval of the IAC; making lactation breaks unpaid for working mothers; allowing distribution of samples in health care facilities, access to health workers by sales and marketing staff, and companies to conduct health professional education and training [86].

In a press release, Representative Lacson-Noel framed her Bill, as fighting “for women’s rights in revising [the] Milk Code” and “to emphasize the importance of informed choice vis-à-vis breastfeeding” [87]. Similar arguments were made in the media by two civil society groups, the first by Women Involved in Nation Building, concerning “the right to information” [88], and the second by Working with Working Mothers describing the Bill as” ...an instrument for women to make informed choices … [that] will empower women and help them make educated choices in health and nutrition”, [89]. In a letter addressed to supporters of the Bill, Rep. Lani Mercado-Revilla supported the pro-breastfeeding position of DOH/WHO/UNICEF, but was;

… also not blind to the plight of individuals who belong to the milk industry and thus … willing to reduce the current ban on advertisement or promotions … [of] between “zero and 36 months” to less than the current period but not less than 12 months [84]

The Secretary of the Department of Trade and Industry wrote to the Chairman of the Committee of Health supporting the consolidated Bill, voicing opposition to any prohibition on the “advertising, promotion, marketing and sponsorship” of BMS, and citing the objective of the Consumer Act to “provide information and education to facilitate sound choice and the proper exercise of the rights of the consumer” [90]. The letter stated any such prohibition infringed upon;

… the fundamental right of consumers, particularly lactating mothers, to information and freedom of choice. Freedom of choice is a basic right, not just as being a consumer but as an individual …

Echoing the statement made by the Department of Trade and Industry back in 2006, the letter further stated that;

… state policies must be liberalized to give industry players, local or foreign, the right to promote their products. Moreover the benefits derived from the performance of the...industry in terms of government revenues and employment opportunities cannot be overemphasized. The proposed restrictions may impact on the sector which employs a substantial number of...workers [90]

In September 2013, a two-part legal opinion, published by a US law firm argued “… the Philippine breastmilk substitute and breastmilk supplement marketing framework … violates the Philippines’ obligation to ensure compliance with the [WTO] Agreements”, which followed a similar legal opinion published in relation to a draft Marketing Code in Hong Kong [91].

These attempts at influencing and weakening the Milk Code and other related policy measures were ultimately unsuccessful. The Memorandum issued by the DOH was sustained, and the Milk Monster Bill failed to pass in the House of Representatives. One informant noted, however, that this was a ‘close call’. The Bill was well advanced in the legislative process (the third reading) before key UN agencies and civil society groups became aware of it. Many initial sponsors of the Bill withdrew their support once they were made aware that it was potentially harmful to maternal, infant and child health.

#### Third major period of CPA, ~ 2010 onwards – crisis marketing and leveraging partnerships

This section demonstrates how more recently, the industry has engaged in ‘crisis marketing’, and leveraged partnerships as a strategy for influencing government agencies and decision-makers.

The Philippines has among the world’s strongest policy frameworks for IYCF in emergencies (described in the following section), including prohibitions on BMS donations. Yet one long-standing challenge has been the frequent occurrence of donations during and in the aftermath of major emergencies, which can contribute to infant malnutrition and death. This is evidenced by a study showing a remarkable rise in BMS donations during the Covid-19 pandemic. Between January 2019 and July 2020, there were 291 reported violations of the countries ‘Milk Code’ legislation, compared with just 70 in 2019, of which 235 (81%) were related to donations of BMS products [92].

Nestlé claims it does not donate BMS during emergencies, yet it is a member of IPNAP, an organization that has actively pursued donation-giving. For example, in August 2012, IPNAP prepared a Memorandum of Agreement (MOU) with the DOH, to allow product donations for children over 6 months in emergencies, and products for infants under 6 months upon the request of the DOH [93], a proposal the DOH rejected following intervention by advocates.

Furthermore, Nestlé continues its practice of corporate philanthropy during emergencies, through large-scale donations of branded ultra-processed food products to local government units and humanitarian NGOs. For example, during the Covid-19 crisis Nestlé donated ‘Kasambuhay’ (or ‘lifetime’) packages to one million families nationwide, excluding CMF products covered by the Milk Code, but including Bear Brand milk formula for older children, a product outside of scope [94]. Earlier, in 2012, the DOH issued a cease and desist notice to Nestlé Philippines for distributing BMS after major flooding [95].

A salient feature of the Milk Monster Bill described earlier was the relaxation of restrictions on BMS donations. Furthermore, in 2020, House Bill 6137 entitled *An Act Encouraging Corporate Social Responsibility, Providing Incentives Therefor*, was re-introduced into the Committee on Trade and Industry with a new provision stating;

All business organizations are allowed to donate products and services under their CSR-related activities for disaster relief and assistance, in accordance with the regulations … issued by the appropriate government agency. All existing law and regulations restricting or prohibiting the right of local government units under a state of calamity and/or during a national emergency to solicit or accept any donation of products and services under the CSR-related activities … are hereby amended [96]

We did not find evidence linking IPNAP activities directly with the proposed Bill. However, if the Bill were to be enacted, this provision would have effectively negated existing legislated prohibitions on BMS donations during emergencies [97].

From 2010 onwards, we observed the formation of various partnerships between milk formula manufacturers and government entities. That year, Mead Johnson promoted its ‘Feeding hope to an impoverished world’ initiative, in partnership with the Department of Social Welfare and Development and Kabisig ng Kalahi Foundation, an NGO established to ‘assist in providing children from poor communities with their nutritional needs’ [98]. In 2012, IPNAP attempted to establish the aforementioned MOU with the DOH [82]. Then in 2013, the Food Nutrition and Research Institute, the Government agency that conducts the country’s National Nutrition Survey, signed an MOU with BMS manufacturers for the development of a six-module training manual in support of *Sulong Pinoy*, a programme to educate local government units about nutrition and maternal health [82].

IPNAP also recruited prominent former-government officials. In 2018, it established an ‘ethics committee’ of prominent individuals, including former Health secretary and Milk Code co-author Dr. Carmencita Reodica, former Health undersecretary Dr. Margarita Galon, and former Trade undersecretary Victorio Mario Dimagiba [99].

IPNAP’s lobbying continued. On March 22, 2016, a letter to the Secretary of Health outlined its position and recommendations on the Philippine response to new WHO draft Guidance on Ending the Inappropriate Promotion of Foods for Infants and Young Children. This stated the new Guidance should be delayed until revision of the Codex Standard on Follow-up Formula was finalized, that a proposed scope of 0–36 months for defining BMS was inappropriate, that adherence to the Guidance on cross-promotion would infringe on manufacturer’s intellectual property rights, and a prohibition on sponsoring health professional and scientific meetings should not be imposed [100]. A letter on November 19, 2020, expressed IPNAP’s interest in working with the DOH in formulating a position on agenda item 15.2 (Maternal, infant and young child nutrition) at the 73rd World health Assembly, and requested clarification of rules set out in the DOH’s new Administrative Order (AO) 2006–0012 concerning digital marketing of products within scope of the Milk Code [101].

Another letter, dated December 15, 2020, outlined IPNAP’s detailed position on the clarificatory guidance on the RIRRs, as set out in AO 2006–0012. This included inter alia that prohibiting health professional training went beyond the initial provisions of the Milk Code law, and prohibiting the use of brand names and trademarks ‘restricts the use of intellectual property rights’. Furthermore, that ‘parents classes’ should not be deemed ‘health facilities’, and ‘marketing materials/services’ should not extend to website advertising, as not all websites function to market products. This also expressed IPNAP’s position on AO 2012–0027 referring to ‘The Inter-Agency Committee (IAC) Guidelines on the Exercise of their Powers and Functions’, stating its opposition to ‘sponsorship and product research’ as being within the IAC’s purview, and the IAC’s consideration of ‘mandatory standard messaging’ as limited to labels and packaging only [101].

#### Emergence of a world-leading policy framework to promote, support and protect breastfeeding

Despite the sustained corporate political activity of the baby food industry described above, rising breastfeeding rates from the early-1980s onwards has resulted, at least to a significant extent, from strengthening political commitment, and the development of a national policy framework to protect, promote and support breastfeeding.

The Philippines began the post-WW2 era with a strong commitment to population development, with explicit pro-nutrition policies initiated by the government since at least 1947. These efforts gained momentum in 1974, when President Ferdinand Marcos adopted Presidential Decree No. 491 declaring nutrition “… a priority of the government to be implemented by all branches of the government in an integrated fashion” [102]. This established a National Nutrition Council (NNC), under the Office of the President, to develop policy and coordinate action on nutrition, including representatives from multiple government agencies and sectors, professional associations, and the private sector. That same year the World Health Assembly (WHA), noting a precipitous decline in breastfeeding in many countries, urged Member states to review BMS-related marketing activities and introduce appropriate counter-measures, including advertising codes and legislation as needed [103].

In May 1981, *The Code* was adopted by the WHA, with 118 Member states voting in favour, three abstaining, and the US the single vote against. That same year, to give immediate effect to *The Code*, the Department of Health (DOH) established a national Code to regulate BMS marketing in the country, and called upon companies for voluntary compliance. Public health facilities were directed to remove commercial infant feeding promotional materials. The National Coalition for the Promotion of Breastfeeding (NCPB) was formed, and began advocating for the full adoption of *The Code* into national law. In 1983, the NCPB joined with the DOH to form the National Movement for the Promotion of Breastfeeding, housed within the DOH, with members from 39 government agencies, 14 non-governmental organizations (NGOs) and 25 other institutes and organizations, with technical and administrative support from UNICEF and WHO. In 1984, medical schools incorporated breastfeeding into curricula, and mass-media campaigns to promote breastfeeding began.

In 1986, the Philippines adopted a new Constitution, recognizing the State’s obligation to ‘ … protect and promote the right to health of the people and instil health consciousness among them’, and ‘ … establish and maintain an effective food and drug regulatory system … responsive to the country’s health needs and problems’ [28]. By the legislative powers granted under the Constitution, President Corazon Aquino promulgated Executive Order No. 51 (EO51), to establish the National Code of Marketing of Breastmilk Substitutes, Breastmilk Supplement and Other Related Products (the *Milk Code*) into national law [104]. This incorporated many provisions of *The Code* to become one the world’s strongest breastfeeding protection laws. It empowered the DOH to promulgate implementing rules and regulations as needed to update and effectively implement the Milk Code, and established an Inter-Agency Committee (IAC), comprising the Departments of Health, Trade and Industry, Justice, and Social Welfare and Development, to review, authorise or prohibit advertising, promotional and other materials within scope.

The DOH is legally mandated and empowered to implement the Milk Code. However, its reach extends to its regional offices only, and the decentralised structure of the Philippines health system poses a key challenge for implementation, dependant as it is upon the leadership and resourcing of local government units, which typically have limited numbers of community health and development workers. Limited awareness of the Milk Code among legislators, government agencies and local government units, within the private sector, and among the wider public is a key challenge for its implementation, with many violators citing ignorance of the regulations. Monitoring and enforcement has typically relied on letters submitted to the DOH, which it then investigates and responds to, a process described as ‘paper-based and bureaucratic’. More recently, the DOH collaborated with the World Vision Development Foundation to establish a ‘crowd-sourced’ digital reporting system, using web and text-message based technologies.

In August 1990, the Philippines ratified the Convention on the Rights of the Child (CRC), recognising the right of all children to the highest attainable standard of health, and specifically under Article 24, the right to optimal nutrition, including breastfeeding. The Philippines, as a State Party to the CRC, was required to align national policies and laws with its provisions, and to protect its citizens from the unlawful infringement of such rights. It was required to report on its progress to the UN Committee on the Rights of the Child, which it has done on four occasions since [105]. In September 2004, the DOH initiated a process to strengthen the Milk Code, through revised Implementing Rules and Regulations (RIRRs) [54], which were adopted only after a two-year delay in May 2006, due to sustained industry interference with the process (described later in this manuscript). In 2005 and 2009, the UN Committee on the Rights of the Child expressed serious concern on the country’s low prevalence of breastfeeding, recommending necessary measures are taken to effectively implement the Milk Code and RIRRs [106, 107].

Other important components of the country’s breastfeeding policy framework include the 1992 Rooming-In and Breast-feeding Act (Republic Act No. 7600), which implemented the 1991 standards established by the UNICEF/WHO Baby Friendly Hospitals Initiative [108]; the 2009 Expanded Breastfeeding Promotion Act (RA10028), which required health and non-health facilities to establish lactation stations, and established a ‘working mother-baby’ certification scheme [109]; the 2013 Promote Good Nutrition program, with the aim to enhance people’s knowledge on nutrition, including the promotion of breastfeeding [110]; the 2018 Expanded Maternity Leave Law (RA11210), providing 105 days of paid maternity leave for working mothers [111]; and the 2018 Kalusugan at Nutrisyon ng Mag-Nanay Act (RA11148), to scale-up early-life nutrition intervention programmes, including integration into national and sub-national government development plans. The scope of products covered in RA11148 aligned with the WHO definition of BMS for children aged 0–36 months [112]. Regular nationally representative surveys collect data on infant and young child feeding indicators.

Importantly, given its location in both the Pacific typhoon belt and earthquake-prone Pacific Rim, the Philippines is among the world’s most disaster-prone countries. Coordinating emergency aid and health services across the country’s island populations is extremely challenging. The Philippines has a world-leading system for managing IYCF during emergencies [113], mainly through multi-agency humanitarian coordination platforms, specifically, the National and sub-national Nutrition Clusters. The Nutrition Cluster includes UNICEF as co-lead, international NGOs and local civil society groups. In addition to coordinating emergency nutrition interventions, cluster members also halt prohibited donations of BMS, document and report violations, and support information and advocacy initiatives to guide relief efforts of the private sector. Cluster members’ further support policy discussions at local and national levels, in their varying technical capacities, and knowledge of the national and international Codes.

#### Public health resistance – the role of breastfeeding coalitions and civil society mobilization

The political commitment for breastfeeding described above, has at least to a significant extent, resulted from the mobilization of breastfeeding coalitions, civil society groups and mothers, including organized resistance against the baby food industry.

Members of the National Movement for the Promotion of Breastfeeding (NMPB) mentioned earlier, have consistently responded to the corporate political activities described earlier, including through issuing joint statements, regular media engagement, technical collaboration to counter industry statements, organizing forums and cross-governmental advocacy. The focal point has been a long-standing alliance between the DOH, UNICEF and WHO under the leadership of the Secretary of Health and undersecretaries, key individuals within the UN agencies, as well as certain members of Congress.

For example, in response to the Milk Monster Bill, the UN agencies were proactive in mobilizing partners and forming an evidence-based position, including through a joint technical note, which helped to guide similar actions by civil society groups and other stakeholders. The UN Rapporteur on the Right to Food, Jean Ziegler, also made timely interventions, including denouncing the public relations campaigns and lobbying of PHAP. Ziegler, described the PHAP media campaign in 2007 as “misleading, deceptive, and malicious in intent” [114], and wrote letters to the Chief Justice of the Supreme Court, emphasising the alignment of the RIRRs with The Code and international human rights instruments.

Key actions by other coalitions and groups were also evident. For example, the Save the Babies Coalition, established by the IBFAN-associated NGO Arugaan (meaning ‘to nurture’) and breastfeeding champion Ines Fernandez [115], has consistently responded to industry attempts to weaken the Milk Code, including a 1000 Breastfeeding Defenders initiative, an ‘inter-generational intervention’ protest during the Supreme Court case, and protest events outside Parliament, company offices and elsewhere. Arugaan further established a community support system of peer-counsellors for breastfeeding mothers, and led advocacy efforts to protect and support the rights of working mothers. Nationwide mass-breastfeeding events have also been regularly convened [47, 116–118].

Coalitions have emerged in response to specific issues. For example, several were convened against the Milk Monster Bill in 2013, with focal points in the UN system, civil society organizations and medical professional societies. A large group of health professionals, NGOs and others drafted and signed a joint position paper opposing the Bill. Newer advocacy groups have emerged. The Philippines Coalition of Advocates for Nutrition Security (PhilCan), for example is the convening organization for the Scaling Up Nutrition (SUN) Civil Society Alliance, with a broad membership responsive to various nutrition advocacy needs, including breastfeeding. Several informants stated the importance of these newer groups engaging with more established ones to gain ‘institutional memory’, as they may not always fully understand recurrent Milk Code issues.

Importantly, international NGOs and networks have played key roles. The International Baby Food Action Network (IBFAN) and World Alliance for Breastfeeding Action (WABA) have at times generated international and national media attention through various norm-promotion activities (e.g. country visits, appearances on national television), ‘naming and shaming’ companies by reporting on Code violations, campaigning against industry attempts to weaken the Milk Code (e.g. letter writing, petitions, newsletters), and ‘shareholder activism’ at Nestlé’s annual general meetings in Switzerland [119]. Save the Children, World Vision, Alive & Thrive and PLAN International have also provided important norm-promotion, technical support and advocacy functions. Frequent articles in national and international media, have generated public attention to contests over the Milk Code.

## Discussion

In this study, our aim is to describe and understand the power of the baby food industry, and in particular the strategies it has used to shape the Philippine first-food system, to drive and sustain milk formula consumption. We also consider how breastfeeding coalitions, advocates and mothers resisted, and in many instances overcame, the baby food industry’s influence. Our investigation reveals several thematic insights.

### Breastfeeding, first-food systems and corporate power

#### The power of marketing

As in many other countries, the decline in breastfeeding in the Philippines in the mid-to-late 20th Century closely linked with widespread and intensive BMS marketing. Such marketing is still prominent in the country today. To sustain high levels of milk formula sales in the Philippines, the baby food industry has since the mid-1980s, more aggressively promoted products for older infants and young children, which are now a major feature of the country’s first-food system [4]. This is a strategy adopted by the industry worldwide to ‘widen the scope’ of its market, as regulations have tightened on the marketing of infant formulas [14].

This has included ‘medical marketing’ techniques, including extensive engagement with health professionals, alongside direct-to-consumer advertising and widespread product distribution [14]. Recruiting prominent medical professionals as members of the ‘speakers bureau’ or ‘key opinion leaders’ is a common strategy used by pharmaceutical companies [120, 121]. Nestlé’s stated opposition to prohibitions on health professional engagement and education during the RIRRs drafting process underscores how important medical marketing is to sustaining CMF sales. ‘Crisis marketing’ is also evident in the Philippines [14], strongly indicated by the recent surge in BMS donations during the Covid-19 epidemic [92].

#### The power to market

The baby food industry has not only made large investments in the marketing and inappropriate promotion of its products. Our findings also show that, in order to support and sustain such marketing, it has since at least 2004, implemented sustained political strategies to prevent, delay or weaken the country’s national IYCF policy framework, and especially the Milk Code. The financial cost of implementing these strategies is very likely negligible for the baby food industry, considering the US$840 million value of the Philippine CMF market.

##### Establishing front groups and political distancing:

We show that two industry ‘front groups’ have been instrumental in implementing these strategies. The first PHAP, representing US baby food and pharmaceutical corporations, engaged in aggressive action against the Milk Code, including through highly-visible media and litigation. More recently IPNAP, which appears to be led by Abbott and Nestlé, but also represents a number of other transnational CMF manufacturers, has operated in less visible and more covert ways. Internationally, the industry has used such front groups since at least the 1970s, in a strategy that allows the corporations to ‘politically distance’ themselves, and thereby avoid the reputational damage that results from lobbying [14]. Although Nestlé attempted to distance itself from the more nefarious activities of PHAP, it then joined forces with the US companies in IPNAP.

##### Mobilizing a global corporate influence network:

Lobbying by the baby food industry has occurred at the highest level in the Philippines, including directly to the President, members of Congress, and government officials in the health, trade and industry sectors. Importantly, we also show how the industry mobilized and coordinated a much-wider global lobbying network to amplify this influence [14]. This included the engagement of US government diplomats, the US Chamber of Commerce, and another US-based ‘infant nutrition’ front group, which in-turn lobbied the Philippine Embassy and the UNICEF international and regional headquarters. The ‘economic diplomacy’ by the US government on behalf of the industry is consistent with experiences in Viet Nam, Hong Kong, Thailand and elsewhere [14], suggesting this is a major challenge for implementation of The Code across the region.

##### Leveraging market power and international capital mobility:

Prominent and consistent messages used by the industry emphasised the jobs and investments it provides in the Philippines, alongside threats to investment and trade implications if regulations were imposed. These ‘economic importance’ arguments are representations of the significant ‘structural power’ that transnational corporations often hold over governments, through their international capital mobility, a form of power that has been amplified by economic globalization [122]. The Government’s own Department of Trade & Industry used similar ‘productivist’ arguments, emphasising the importance of market liberalization, and the potential damage regulations posed to the infant formula sub-sector, employment and government revenues.

##### Emphasising choice and women’s empowerment:

We also observed a set of messages used in support of regulatory changes favourable to industry, or against those that were unfavourable, that framed BMS marketing as a social good in terms of ‘consumer education’, and the regulation of such marketing as a threat to ‘women’s empowerment’, including the mother’s ‘right to choose’, the ‘right to information’, the ‘rights of the consumer’, and ‘informed choice’. We could not ascertain whether the use of these frames by certain civil society groups and members of Congress were unwittingly adopted with good intention, or whether this was the result of direct corporate engagement to influence wider public debate. This frequent use of rights-based messaging contrasts with the actual human rights obligations of the Philippines, including as a state party to the Convention on the Rights of the Child. It also contrasts with the ‘principles-based messaging’ and ‘moral power’ of civil society groups.

##### Litigation and the threat of trade arbitration:

The most brazen action by industry was the legal action PHAP initiated against the DOH in the Supreme Court, a move that resulted in a temporary restraining order (TRO) and delay in the strengthening of the country’s Milk Code. Arguably this represents a form of structural violence against the child [123], given an estimated 27 infants were at risk of dying for every day the TRO was in place, or 10,746 deaths in total over the 398 day period the TRO was in place. Messaging also emphasised the Philippines obligations under the WTO’s TBT and TRIPS agreements. Similar arguments concerning infringements on the intellectual property rights of corporations have been made for decades, and although no BMS-related trade arbitration in the WTO or elsewhere has ever eventuated [124], this may still have a ‘chilling effect’ on national regulators [14].

##### Stealth legislation:

The Milk Monster Bill, if passed, would have significantly weakened the country’s breastfeeding policy framework and Milk Code. Yet the legislative process was well advanced before the dangers of this Bill became apparent to public health groups. This indicates how major legislative change favourable to industry can proceed without scrutiny, and emphasises the need for constant vigilance to protect and sustain the country’s breastfeeding policy framework. That many initial supporters of the Bill withdrew their support once the extent of negative implications became known, suggests the framing of the Bill in terms of women’s empowerment and choice, may have helped industry mobilize support from legislators without their deeper scrutiny.

##### Forging partnerships and government connections:

The industry appears to have evolved its strategy more recently by forging partnerships with government agencies, and recruiting prominent former government officials. This strategy may not only provide the organization with ‘inside’ connections to regulators, but also enhances its legitimacy as a responsible corporate actor and policy partner, something which can lead eventually to so-called ‘regulatory capture’ [125]. The recruitment of a former DOH employee and Congressman to lead the IPNAP front group is clear indication of the ‘revolving door’ strategy used by the baby food industry for decades [14].

### Public health resistance – the power of breastfeeding coalitions, advocacy groups and mothers

Despite the size, power and resources of the baby food industry, and its government and business allies, breastfeeding coalitions, advocates and mothers have successfully generated political commitment for a world-leading breastfeeding policy framework and Milk Code in the Philippines.

#### The power of breastfeeding coalitions

The DOH with the support of cross-sectoral government agencies, UNICEF, WHO and national and international NGOs, developed a powerful lobby for breastfeeding that frequently and effectively countered industry influence. This underscores the importance of sustaining and indeed strengthening this coalition under the leadership of the DOH, and continuing financial support for the norm-promotion, technical and advocacy work of UNICEF, WHO and civil society partners in the country. Few studies have investigated how breastfeeding coalitions emerge, become effective and sustain themselves over time. Studies on related issues, including child and maternal health [126, 127], and nutrition [128], find that cohesion, leadership, strategic capacity, and resourcing are important, among other context-dependant factors.

#### Civil society mobilization and transnational activism

Civil society groups, breastfeeding champions, health professional societies, journalists, and at times large numbers of mothers themselves, have mobilized collectively in response to industry attempts to weaken the Milk Code. Such mobilization is recognised as an enabling factor for breastfeeding and nutrition improvement within countries, especially for awareness-raising, strengthening accountability and giving voices to mothers and children [129, 130]. Importantly, the power of this grass-roots mobilization was amplified by a ‘transnational advocacy network’ centred on IBFAN, which helped generate international attention and pressure, and at times provided direct advocacy support within the Philippines itself. Such networks are distinguishable by their shared values and principled ideas, and ability to mobilize members across borders [131].

#### The combined technical and moral power of breastfeeding advocacy

Breastfeeding coalitions and advocates have used compelling ‘evidence-based’ arguments for the protection, promotion and support of breastfeeding in the Philippines, drawing from well-established international standards and ‘consensus’ scientific knowledge. However, arguably of equal importance is the principles-based messaging also frequently used, reflecting widely held beliefs concerning the rights of the mother and child, and the protection of the innocent from commercial harm. On occasion, such arguments directly emphasised the conflict between industry objectives of profit-maximisation with the health of mothers and children, or in short the juxtaposition of ‘property rights and human rights’. This has arguably helped the issue reach an ‘emotional plane’ in the Philippines, and maintained the issues salience [9].

### Strengths and limitations

A major strength of this analysis was the remarkable amount of documentary evidence we sourced, including from our informants. The process tracing method enabled rich description of unfolding events, and how industry practices changed over time. We applied a robust case study design and process tracing method, triangulating our data where possible. There were several limitations. The single case study makes generalising to other countries difficult. Our study has not considered the broader challenges involved with monitoring and enforcing the Milk Code, although our informants raised this as a crucial challenge for countering the power of marketing. The corporate political activities we report began in 2004, although such activities likely started much earlier, given the long history of the baby food industry in the country. We report ‘crisis marketing’ as becoming evident during the period from 2010 onwards, however, reports of such marketing have been made much earlier than this elsewhere. We have not reported on levels of financing for breastfeeding, even though this is an important indicator of political commitment for the implementation of the country’s breastfeeding policy framework. We have also not considered the rise of commercial complementary foods in the country, although these foods are now a significant share of children’s diets. These are topics for future research.

## Conclusion

We find that the decline in breastfeeding and the rise in CMF consumption in the Philippines has associated with the intensive marketing practices of the baby food industry, and that such practices are in themselves a powerful way in which the industry shapes first-food systems. Arguably of equal importance, we also show how this industry uses a number of political strategies to protect and sustain its CMF market, through actions against the country’s breastfeeding policy framework, and especially the Milk Code. Overall, our findings suggest that these corporate political activities are a major impediment to worldwide implementation of The Code into national laws, and that new modalities of public health action are needed to curtail this corporate power over first-food policy and systems regulation. There is a demonstrated need to safeguard the development of policies to protect, promote and support breastfeeding from the baby food industry, as many countries have already done for tobacco control.

Our findings also suggest the more recent resurgence in breastfeeding in the Philippines is, at least to a significant extent, the result of rising political commitment for breastfeeding, and the emergence of a comprehensive breastfeeding policy framework, including among the world’s strongest breastfeeding protection laws. However, our findings highlight the need for continued vigilance in order to protect this policy framework, and the importance of sustaining and indeed strengthening the country’s breastfeeding coalition under the leadership of the DOH, including coordinated efforts with regional offices, partner government agencies (who are also members of the National Nutrition Council), local government units and civil society groups. This also includes ensuring resources and dedicated personnel for UNICEF, WHO and civil society partners in the country to continue their technical support, norm-promotion and advocacy work.

Ultimately, such actions will help to advance the greater right to health and nutrition of the people, and most of all the rights of Filipino mothers and children, over vested commercial interests.

## Supplementary Information

**Additional file 1.** Text S1 – Theoretical framework.

## Data Availability

All data generated or analysed during this study are included or cited in this published article and its supplementary information files. The information contained in this manuscript has been obtained from sources believed to be reliable. However, any potential interpretation of the findings as making an allegation against a specific named company or companies would be incorrect and misleading.
