# Supplementary material for: Breastfeeding, first-food systems and corporate power: a case study on the market and political practices of the transnational baby food industry and public health resistance in the Philippines
Source: Global Health. 2021 Oct 26;17:125. doi: 10.1186/s12992-021-00774-5 (PMC8547294; doi:10.1186/s12992-021-00774-5)
Supplement: Supplementary file 1 — Additional file 1. Text S1 – Theoretical framework. [file 12992_2021_774_MOESM1_ESM.docx]

### Text S1 – Theeoretical framework

We have defined and descrirbed the main components of first-foods systems in our earlier work (Baker, 2020; Baker, Melo, et al., 2020). To understand the power of the baby food industry to shape first-foods systems, we integrated concepts from the commercial determinats of health (CDOH) and political economy of food systems literatures (Table 1) (Clapp & Fuchs, 2009; Clapp & Scrinis, 2017; Fuchs et al., 2016; Madureira Lima & Galea, 2018; McKee & Stuckler, 2018).

**Table 1.** Theoretical framework used to understand corporate power and guide the study

| **Source of power** | **Forms of power** | **Actors & relations of power** | | | |
| --- | --- | --- | --- | --- | --- |
|  |  | **Market actors** | **State actors** | **Civil society** | **Experts** |
| Material assets and resources | Instrumental |  |  |  |  |
|  | Structural |  |  |  |  |
|  | Discursive |  | Market & political practices | Market & political practices | Market & political practices |
| **Contextual factors** (amplify or constrain power)  Political, legal, technological, economic, and socio-cultural | | | | | |

First, we defined corporate actors, something often missing in CDOH scholarship. ‘Big Tobacco’ is often used as a collective term for the world’s largest tobacco manufacturers. Similarly, we used ‘Big Formula’ to refer to the corporations that manufacture and distribute BMS on an industrial scale, most but not all, being transnatioanl corporations with a market presence in two or more country markets. We also viewed each corporation as anchored in their country of origin, and hence as identifying with nationally-derived cultures, operational structures and relationships with their home country governments (Mikler, 2018; Wilks, 2013). The ‘baby food industry’ comprises Big Formula at its core, but also the dairy industry and other input suppliers, retailers, advertising agencies, and various other commerical entities who profit from BMS (Coriolis, 2014; Palmer, 2009).

Executives and senior management run corporations, with a fiduciary duty to maximise profit, and through sustained profit, generate returns to shareholders (the owners) (Mikler, 2018; Wilks, 2013). To realise this interest, every effort is made within the legal constraints the corporation operates under, and sometimes beyond these constraints, to externalize as much of its costs of production as possible. The functioning of the market economy ensures these costs (or in economic terms ‘externalities’) are in the public domain, and so must be addressed by governments, or absorbed by social groups (e.g. higher morbidity, and health care costs) and/or the environment (e.g. water pollution or greenhouse gas emissions from dairy production). In pursuit of their interest, corporate actors seek to minimise conflict, neutralise or co-opt other societal actors , be they market (e.g. consumers, competing firms or suppliers), state (e.g. governments and inter-governmental organizations), civil society (e.g. non-governmental organizations, social movements and the media), and expert (e.g. scientists, academics and health professionals) actors (Sethi, 2012).

Sholarship on the tobacco, alcohol and ultra-processed food industries often refers to a set of market and political practices (i.e. applied strategies and tactics) used to influence other actors within the system (Madureira Lima & Galea, 2018; Mialon, Swinburn, & Sacks, 2015; Moodie et al., 2013). We organized these same practices under several overlapping and reinforcing concepts of power.

Arguably, the main source of corporate power is material, referring to the assets and resources acquired by corporations over time (Fuchs, 2007; Fuchs et al., 2016). With regards to Big Formula, we considered *inter alia* their sales revenues, profits, finance, productive assets (e.g. factories), human resources, trademarks and proprietary technologies among others. As corporations grow and globalize, these accumulating assets and resources can be readily converted into instrumental, structural and discursive forms. Instrumental power is the power to influence others directly (Clapp & Fuchs, 2009; Fuchs, 2013; Fuchs et al., 2016). For example, we anticipated that corporate executives may be members of elite social networks, with direct access to political leaders and government officials. Furthermore, that Big Formula uses its resources (and also ‘pools resources’ across the industry) to hire lobbyists, lawyers and public relations firms, make political donations, recruit former governmental officials, finance front groups and think tanks, form business coalitions, employ large sales forces to engage health professionals, and so on.

Structural power is the power to shape agendas and control the behavioural options available to others, without taking direct action (Clapp & Fuchs, 2009; Clapp & Scrinis, 2017; Fuchs, 2013). For example, governments might make regulatory concessions to attract (or retain) the investments and employment opportunities Big Formula provides. In a strategy known as policy substitution, corporations might adopt voluntary private standards to delay or even replace regulation by the state; or support public-private partnerships (PPPs), that expand corporate influence in defining policy agendas and decision-making. As markets become more consolidated, Big Formula might exert greater power over suppliers to reduce costs (i.e. oligopsonistic power), control the product types and prices available to consumers (i.e. oligopolistic power), and thereby maximise its profit margins. Discursive power is the power to shape attention, influence (or supress) knowledge and evidence, and frame debates (Clapp & Fuchs, 2009; Clapp & Scrinis, 2017; Fuchs, 2013). It is the power to socialise others, often unconsciously, into accepting certain problem interpretations and behaviours as normal, acceptable or socially desirable. To this end, we anticipated that Big Formula might finance public relations initiatives, attempt to shape scientific processes and wider knowledge environments, and engage in sophisticated forms of marketing.

We viewed these forms of power as interacting. For example, to counter regulatory threats, lobbyists may coordinate their discursive strategies across multiple decision-making spaces simultaneously; private standards can be both a form of structural power by substituting for regulation by the state, and discursive by portraying corporations as responsible social actors; marketing not only influences and drives consumer behaviour, but also socialises health professionals, policy-makers and others into adopting pro-industry beliefs.

Contextual factors support or constrain corporate power, including the political, legal, technological, economic, and socio-cultural structures and systems in which they operate (McDonough, Reich, & Kotz, 2010; Sammut-Bonnici & Galea, 2014). For example, we anticipated that trade and investment liberalization has enabled Big Formula’s global expansion, including its cross-border supply chains, while the expanding scope and depth of trade agreements has constrained the ‘policy space’ of governments to regulate formula markets within their borders (Baker, Machado, et al., 2020; Milsom, Smith, Baker, & Walls, 2020). Inadequate paid maternity leave entitlements in many countries, enables Big Formula’s power, by making formal maternal employment less compatible with breastfeeding. We viewed a major constraint on the power of Big Formula as the norm-promotion and accountability work of civil society groups (e.g. IBFAN), international organizations (e.g. WHO, UNICEF) and others (Baker, Melo, et al., 2020; Keck & Sikkink, 2014).

**References**

Baker, P. (2020). Breastfeeding, first food systems and corporate power. *Breastfeeding Review, 28*(2), 33-37.

Baker, P., Machado, P., Santos, T., Sievert, K., Backholer, K., Hadjikakou, M., . . . Scrinis, G. (2020). Ultra-processed foods and the nutrition transition: Global, regional and national trends, food systems transformations and political economy drivers. *Obesity Reviews*.

Baker, P., Melo, T., Augusto Neves, P., Machado, P., Smith, J., Piwoz, E., . . . McCoy, D. (2020). First-food systems transformations and the ultra-processing of infant and young child diets: a synthesis of data and literature on the determinants, dynamics and consequences of the global rise in milk formula consumption. *Maternal & Child Nutrition, 17*(2), 1-18.

Clapp, J., & Fuchs, D. (2009). Agrifood corporations, global governance, and sustainability: a framework for analysis. In D. Fuchs & J. Clapp (Eds.), *Corporate power in global agrifood governance* (pp. 1-25). Cambridge: MIT Press.

Clapp, J., & Scrinis, G. (2017). Big food, nutritionism, and corporate power. *Globalizations, 14*(4), 578-595.

Coriolis. (2014). *Understanding the infant formula value chain*. Retrieved from <https://coriolisresearch.com/pdfs/coriolis_dairy_infant_formula_value_chain.pdf>

Fuchs, D. (2007). Exploring the Role of Business in Global Governance. In *Business Power in Global Governance*. Boulder, Colorado: Lynne Rienner.

Fuchs, D. (2013). Theorizing the power of global companies. *The handbook of global companies. West Sussex: John Wiley & Sons*, 77-95.

Fuchs, D., Di Giulio, A., Glaab, K., Lorek, S., Maniates, M., Princen, T., & Røpke, I. (2016). Power: the missing element in sustainable consumption and absolute reductions research and action. *Journal of Cleaner Production, 132*, 298-307.

Keck, M. E., & Sikkink, K. (2014). *Activists beyond borders: Advocacy networks in international politics*: Cornell University Press.

Madureira Lima, J., & Galea, S. (2018). Corporate practices and health: a framework and mechanisms. *Globalization and Health, 14*(21). 10.1186/s12992-018-0336-y

McDonough, T., Reich, M., & Kotz, D. (2010). Introduction: Social Structure of Accumulation Theory for the 21st Century. In D. Kotz, M. Reich, & T. McDonough (Eds.), *Contemporary Capitalism and its Crises: Social Structure of Accumulation Theory for the 21st Century*. Cambridge: Cambridge University Press.

McKee, M., & Stuckler, D. (2018). Revisiting the Corporate and Commercial Determinants of Health. *American Journal of Public Health, 108*(9), 1167-1170. 10.2105/AJPH.2018.304510

Mialon, M., Swinburn, B., & Sacks, G. (2015). A proposed approach to systematically identify and monitor the corporate political activity of the food industry with respect to public health using publicly available information. *Obesity reviews, 16*(7), 519-530.

Mikler, J. (2018). *The political power of global corporations*: John Wiley & Sons.

Milsom, P., Smith, R., Baker, P., & Walls, H. (2020). Corporate power and the international trade regime preventing progressive policy action on non-communicable diseases: a realist review. *Health policy and planning*.

Moodie, R., Stuckler, D., Monteiro, C., Sheron, N., Neal, B., Thamarangsi, T., . . . Group, L. N. A. (2013). Profits and pandemics: prevention of harmful effects of tobacco, alcohol, and ultra-processed food and drink industries. *The Lancet, 381*(9867), 670-679.

Palmer, G. (2009). *The politics of breastfeeding: When breasts are bad for business*: Pinter & Martin Publishers.

Sammut-Bonnici, T., & Galea, D. (2014). PEST analysis. In C. Cooper (Ed.), *Wiley Encyclopedia of Management*. New Jersey: Wiley.

Sethi, S. P. (2012). *Multinational corporations and the impact of public advocacy on corporate strategy: Nestle and the infant formula controversy* (Vol. 6): Springer Science & Business Media.

Wilks, S. (2013). The national identity of global companies. *The handbook of global companies*, 35-52.
